# Electrospun Methacrylated Gelatin/Poly(L-Lactic Acid) Nanofibrous Hydrogel Scaffolds for Potential Wound Dressing Application

**DOI:** 10.3390/nano12010006

**Published:** 2021-12-21

**Authors:** Mingchao Sun, Shaojuan Chen, Peixue Ling, Jianwei Ma, Shaohua Wu

**Affiliations:** 1 College of Textiles and Clothing, Qingdao University, Qingdao 266071, China; 15662505526@163.com (M.S.); qdchshj@126.com (S.C.); 2 Shandong Academy of Pharmaceutical Science, Key Laboratory of Biopharmaceuticals, Engineering Laboratory of Polysaccharide Drugs, National-Local Joint Engineering Laboratory of Polysaccharide Drugs, Jinan 250101, China; lpxsdf@163.com

**Keywords:** electrospinning, hydrogel, methacrylated gelatin, poly(L-lactic acid), wound dressing

## Abstract

Electrospun nanofiber mats have attracted intense attention as advanced wound dressing materials. The objective of this study was to fabricate methacrylated gelatin (MeGel)/poly(L-lactic acid) (PLLA) hybrid nanofiber mats with an extracellular matrix (ECM) mimicking nanofibrous structure and hydrogel-like properties for potential use as wound dressing materials. MeGel was first synthesized via the methacryloyl substitution of gelatin (Gel), a series of MeGel and PLLA blends with various mass ratios were electrospun into nanofiber mats, and a UV crosslinking process was subsequently utilized to stabilize the MeGel components in the nanofibers. All the as-crosslinked nanofiber mats exhibited smooth and bead-free fiber morphologies. The MeGel-containing and crosslinked nanofiber mats presented significantly improved hydrophilic properties (water contact angle = 0°; 100% wettability) compared to the pure PLLA nanofiber mats (~127°). The swelling ratio of crosslinked nanofiber mats notably increased with the increase of MeGel (143.6 ± 7.4% for PLLA mats vs. 875.0 ± 17.1% for crosslinked 1:1 MeGel/PLLA mats vs. 1135.2 ± 16.0% for crosslinked MeGel mats). The UV crosslinking process was demonstrated to significantly improve the structural stability and mechanical properties of MeGel/PLLA nanofiber mats. The Young’s modulus and ultimate strength of the crosslinked nanofiber mats were demonstrated to obviously decrease when more MeGel was introduced in both dry and wet conditions. The biological tests showed that all the crosslinked nanofiber mats presented great biocompatibility, but the crosslinked nanofiber mats with more MeGel were able to notably promote the attachment, growth, and proliferation of human dermal fibroblasts. Overall, this study demonstrates that our MeGel/PLLA blend nanofiber mats are attractive candidates for wound dressing material research and application.

## 1. Introduction

Wound dressing materials, such as gauzes, bandages, adhesive films, and hydrogel covers, are widely-employed medical devices to treat various types of open wounds and accelerate their healing [1]. Wound dressing materials should have some significant features and functions, including covering the wound without further contamination, inhibiting the invasion of exogenous microorganisms, and maintaining the appropriate moisture of the environment without the accumulation of fluid [2]. Some previous studies demonstrated that the effective replication of the structure and biophysical properties of the native extracellular matrix (ECM) is a key factor for accelerated wound healing [3,4].

Electrospinning is a simple and versatile method used to directly spin polymeric solutions or melts into nanofibers, with diameters ranging from several to several hundred nanometers, which were at least 100 times thinner than fibers fabricated with traditional melt spinning and solution spinning [5,6,7]. Most recently, electrospun nanofibrous mats have found potential applications as wound dressing materials due to their high surface-to-volume ratio, small pore size, and high porosity [8]. In other words, electrospinning is known to produce an ideal structure with a small pore size and high porosity for wound dressing applications that can not only provide a good barrier but also impart excellent air permeability [9]. More importantly, electrospun nanofibers were found to better mimic the scale and morphology of protein fibrils existed in native ECMs, which could provide beneficial microenvironments for promoting cell attachment, growth, and proliferation, as well as the differentiation of stem cells [10,11].

In the last decade, a variety of natural and synthetic polymers have been successfully electrospun into nanofibers [12,13,14]. Commonly-used natural polymers for the fabrication of electrospun nanofibers include collagen, Gel, chitin, chitosan, and silk fibroin [15,16], whereas synthetic polymers include poly(L-lactic acid) (PLLA), poly(glycolic acid) (PGA), poly(lactic-co-glycolic acid) (PLGA), poly(ε-caprolactone) (PCL), and poly(p-dioxanone) (PPDO) [17]. Gel generated from collagen is a biocompatible and bio-absorbable natural polymer that has been widely utilized for the fabrication of wound dressings [18,19]. Importantly, Gel-based wound dressings exhibit ECM-like hydrogel characteristics, resulting in accelerated wound healing [20]. However, its inferior mechanical properties and significantly fast and uncontrollable degradation rate are the main drawbacks of pure Gel materials. Moreover, pure Gel possesses poor electrospinnability due to its strong hydrogen bonding [21,22]. Most recently, a modified Gel, i.e., methacrylated gelatin (MeGel), was synthesized via the methacryloyl substitution of Gel. The structural stability and degradation rate of MeGel-based hydrogels could be easily controlled by adjusting the UV crosslinking process [23]. Importantly, a MeGel-based hydrogel can maintain its high biocompatibility similar with the unmodified gelation, which was widely employed as cell delivery carriers [24]. Unlike Gel-based natural polymers, PLLA is a biocompatible and biodegradable synthetic polymer that presents high mechanics and electrospinnability but poor hydrophilicity and biological properties [25,26,27]. Therefore, electrospinning a hybrid polymeric system composed with MeGel and PLLA may be a great routine to combine the desirable characteristics and reduce the poor properties of both Gel and PLLA.

In this work, MeGel was firstly synthetized, and then a series of MeGel and PLLA blends with different mass ratios were utilized to manufacture electrospun nanofiber mats. Finally, a UV crosslinking process was employed to stabilize the MeGel component of as-developed MeGel-containing nanofiber mats. The authors of this study systematically explored how the MeGel/PLLA weight ratio affected the morphological, structure, physical, chemical, and mechanical properties of nanofiber mats. Moreover, human fibroblasts were further seeded on different MeGel/PLLA nanofiber mats to preliminarily evaluate their potential as wound dressing materials.

## 2. Materials and Experimental Methods

### 2.1. Materials

PLLA (M_W_ = 100,000 Da) was purchased from Jinan Daigang Biomaterial (Jinan, China). Gel (Type B from bovine skin), hexafluoro-2-propanol (HFIP, Purity ≥ 99.8%), 2-hydroxy-1(4-(hydroxyethyl)phenyl)-2-methyl-1-propanone (Irgacure 2959), and methacrylic anhydride were all purchased from Aladdin Reagent (Shanghai, China). All the polymers and chemicals were used as received without any further purification.

### 2.2. Gel Modification and MeGel Generation

Gel was modified to generate MeGel according to previous reports [28,29]. Briefly, 5 g of Gel were dissolved into 50 mL of distilled water to form a homogeneous solution with a concentration of 10% (*w*/*v*) at 40 °C. Then, 10 mL of methacrylic anhydride were dropwise added in the Gel solution, and a chemical reaction was allowed to occur for 1 h at 40 °C. After that, the obtained MeGel solution was dialyzed for 7 days and frozen at −80 °C overnight. Finally, the frozen MeGel materials were lyophilized at the temperature of about −50 °C and a pressure of about 5 Pa for 7 days to completely remove the solid water, and the as-obtained dry MeGel materials were stored at −20 °C before further use.

### 2.3. Fabrication of MeGel/PLLA Nanofiber Mats

A series of MeGel and PLLA mixtures with different mass ratios, i.e., 1:0, 4:1, 1:1, 1:4, and 0:1, were dissolved into an HFIP solvent to produce five different spinning solutions with a fixed polymer concentration of 10% (*w*/*v*). All these solutions were magnetically stirred at room temperature until homogeneous solutions were obtained. The five different MeGel/PLLA solutions were electrospun into nanofiber mats with a typical electrospinning device. The polymer solution was loaded into a 10 mL syringe with an 18 G blunt-tip needle, and a syringe pump was employed to control the flowing rate of the polymer solution to a fixed value of 0.8 mL/h. The needle was connected with the positive polar of a high voltage supply, and 15 kV of voltage were continuously provided. A grounded aluminum plate was utilized to collect the nanofibers ejected from the needle, and the distance between the collector and needle was maintained at 18 cm. All electrospun nanofiber mats were put in a vacuum-drying chamber for 72 h to remove the HFIP solvent. The MeGel-containing nanofiber mats (MeGel/PLLA = 1:0, 4:1, 1:1, and 1:4) were further soaked in a 5% (*w*/*v*) Irgacure 2959/ethanol solution for 10 min. Subsequently, a UV lamp (OmniCure S2000, Lumen Dynamics, Mississauga, ON, Canada) was utilized to crosslink and stabilize the MeGel component in the nanofiber mats. The stabilized nanofiber mats were washed three times using deionized water to remove the un-crosslinked Irgacure 2959, and then they were put into a −80 °C freezer overnight and lyophilized before characterization.

### 2.4. Material Characterizations

Proton nuclear magnetic resonance (^1^H NMR, Bruker AVANCE III 600 MHz, BRUKER Co, Ltd., Zurich, Switzerland) was utilized to characterize the degree of the methacryloyl substitution of MeGel. We completely dissolved 30 mg of Gel or MeGel in 1 mL of deuterium oxide (D_2_O) at 37 °C. MestReNova software was employed to analyze the data and calculate the degree of methacryloyl substitution according to the following formula [30]:

(1) $$\mathrm{DM}\left(\%\right)=0.3836\times \frac{I_{5.5\mathrm{ppm}}}{I_{0.8\mathrm{ppm}}}\times \frac{100}{0.0385}$$

where DM is the degree of methacryloyl substitution and *I*_5.5ppm_ and *I*_0.8ppm_ represent the integral areas corresponding to the peaks at 5.5 and 0.8 ppm in the ^1^H NMR spectrum, respectively.

Scanning electron microscopy (SEM, TESCAN VEGA3, Brno, Czech Republic) was employed to observe the morphologies of the different nanofiber mats. A common gold coating process was performed before the SEM observation. The distribution of fiber diameter and the average fiber diameter were determined with Image J software (NIH, Bethesda, MD, USA). During the calculation, more than 100 different locations were randomly chosen based on 3 different SEM images for each specimen.

A Fourier-transform infrared (FTIR) spectrometer (Bruker TENSOR27, Borken, Germany) was utilized to characterize the functional groups of the different nanofiber mats. The scanning was carried out at a 4 cm^−1^ resolution in the range of 500~4000 cm^−1^.

The X-ray diffraction patterns of the nanofiber mats with different MeGel/PLLA weight ratios were determined using a Rigaku Ultima IV (Tokyo, Japan) with Cu Kα radiation. The detection was performed with a velocity of 5° (2θ) per minute in the 2θ range of 5~90°.

The surface hydrophilicity and wettability of the mat specimens were measured with a video contact angle machine (XG-CAMD3, Shanghai, China). All mat samples were cut into 10 × 30 mm rectangles and fixed on glass slides. A deionized water droplet with a volume of 2 μL was applied onto the surface of each mesh sample, and the change of water contact angle was recorded with the increase of time until balance was reached.

The swelling behavior of the different nanofiber mats was explored by immersing the dried mat samples into deionized water at room temperature. At predetermined time points, the swollen mat samples were taken out and weighed after the adsorbed water on the surface was wiped off using filter paper. The swelling ratio was calculated with the following formula:

(2) $$\mathrm{Swelling}\;\mathrm{ratio}\left(\%\right)=\frac{W_{t}-W_{0}}{W_{0}}\times 100\%$$

where W_0_ is the original weight of each mat sample in dry state and W_t_ is the weight of the corresponding mat sample in wet state at predetermined time points.

The swelling kinetics of the different crosslinked nanofiber mats were calculated by using the equilibrium swelling ratio ($W_{\infty }$) and the dynamic swelling ratio (W_t_) according to the Formulas (3) and (4), respectively [31,32,33,34,35,36].

(3) $$W_{\infty }=\frac{M_{\infty }-M_{0}}{M_{0}}$$

(4) $$W_{t}=\frac{M_{t}-M_{0}}{M_{0}}$$

where M_0_ is the original weight of each mat sample in the dry state, M_t_ is the wet weight of each sample when the time is t, and $M_{\infty }$ is the wet weight of each sample in the swelling equilibrium status.

The swelling kinetics were further studied by using two classical models. We used Formulas (5)–(7) [37] to explore whether the swelling kinetics of all mat samples fit first-order kinetics model.

(5) $$\frac{\mathrm{dW}}{\mathrm{dt}}=k_{w}\left(W_{\infty }-W_{t}\right)$$

(6) $$\mathrm{In}\frac{W_{\infty }}{W_{\infty }-W_{t}}=k_{w}t$$

(7) $$k_{w}t=-\mathrm{In}\left(1-\frac{W_{t}}{W_{\infty }}\right)$$

where ($W_{\infty }$ − W_t_) represents the remaining swelling capacity and k_w_ is the ratio between swelling ratio and swelling capacity number.

We used Formulas (8) and (9) [38] to explore whether the swelling kinetics of all mat samples fit the Schott second-order swelling kinetic model.

(8) $$\frac{\mathrm{dW}}{\mathrm{dt}}=k_{s}\left(W_{\infty }-W\right)$$

(9) $$\frac{t}{W}=A+\mathrm{Bt}$$

where B is the reciprocal of equilibrium swelling ratio ($W_{\infty }$), $B=\frac{1}{W_{\infty }}$; A is the reciprocal of initial swelling ratio, $A=\frac{1}{\left(\frac{\mathrm{dW}}{\mathrm{dt}}\right)0}$; and k_s_ is the swelling ratio constant of second-order kinetics, $A=\frac{1}{k_{s}W_{\infty }{}^{2}}$.

The air permeability of each mat sample was tested with an air permeability tester (FX3300 IV, TEXTEST Co. Ltd., Schwerzenbach, Switzerland). All the different MeGel/PLLA nanofiber mats were cut into 6 × 6 cm pieces and then put in the air permeability tester. A fixed testing area of 20 cm^2^ and a fixed pressure drop of 200 Pa were utilized for testing.

A universal tensile tester (INSTRON 5965, Norwood, MA, USA) was utilized to characterize the uniaxial mechanical properties of the different MeGel/PLLA nanofiber mats in both dry and wet situations based on the standard (GB/T 3923.1-2013, China). All mat samples were all cut into rectangles with a length of 30 mm and a width of 5 mm. For testing in the wet condition, the samples were immersed into a PBS solution (pH = 7.4) at 37 °C for 5 min. Each mat sample was clamped with a constant distance of 10 mm, and a fixed tensile speed of 10 mm/min was applied to the sample until failure occurred. The Young’s modulus, ultimate strength, and ultimate strain of each sample were calculated in both dry and wet conditions. At least 5 replicates were used for each mat specimen.

### 2.5. Cell Seeding, Culture and Characterization

Human dermal fibroblasts were obtained from the cell bank (Chinese Academy of Sciences, Shanghai, China) and utilized to preliminarily assess the biocompatibility of all crosslinked nanofiber mats with different MeGel/PLLA weight ratios. A medium containing high glucose DMEM (Gibco), 10% fetal bovine serum (FBS, Gibco), and 1% penicillin/streptomycin (P/S, Gibco) was used to culture the cells. In all cell culture experiments, the cells were cultured in a 5% CO_2_ atmosphere at 37 °C. Each mat sample was punched into small discs with a diameter of 10 mm and sterilized in 75% ethanol overnight. A sterilized phosphate-buffered saline (PBS) solution was utilized to wash the sterilized samples 3 times, and the samples were further immersed into the cell culture medium overnight. The human dermal fibroblasts were seeded onto the surface of mat sample with a density of 1 × 10^5^ cells per mat and cultured for 7 days. The medium was changed every two days.

Immunofluorescent staining was employed to visualize the cell morphology and nuclei. The cell-mat constructs were fixed using 4% paraformaldehyde for 4 h after 7 days of culture, penetrated using 0.2% Triton-X100 for 10 min at room temperature, and further blocked using 1% bovine serum albumin (BSA) overnight at 4 °C. IFluorTM 488 phalloidin dye (Yeasen Biotechnology Co., Ltd., Shanghai, China) and 4’,6-Diamidino-2-phenylindole Dihydrochloride (DAPI, Yeasen Biotechnology Co., Ltd., Shanghai, China) were employed to stain the cytoskeleton for 2 h and nuclei for 30 min in the dark, respectively. Finally, a confocal laser scanning microscope (CLSM, Zeiss 900 CLSM, Baden-wurttemberg, Germany) was used to obtain the fluorescent images.

An MTT assay (Sigma) was used to determine the viability and proliferation of human dermal fibroblasts seeded on all different crosslinked mat samples throughout 7 days of culture. At predetermined time intervals, the cell-mat constructs were transferred to 24-well plates and incubated with 1 mL of fresh medium containing 100 μL of a 5 mg/mL MTT solution to form the formazan crystals for 4 h in the dark. Then, the supernatant was gently removed, and the formazan crystals were completely dissolved using 500 μL/well dimethyl sulfoxide (DMSO). Next, 100 μL of the formazan-DMSO solution were removed, and the OD value was read at 490 nm using a microplate reader (Infinite M Nano, Tecan, Mannedorf, Switzerland).

### 2.6. Statistical Analysis

All experiments were performed with at least 3 replicates for each group, and the experimental results were expressed as mean ± standard deviation. Statistical analysis was performed with the Scheffé post-hoc test, and significant differences were determined at *p* < 0.05.

## 3. Results and Discussion

### 3.1. Morphological Characterization of MeGel/PLLA Nanofiber Mats

**Figure 1:**
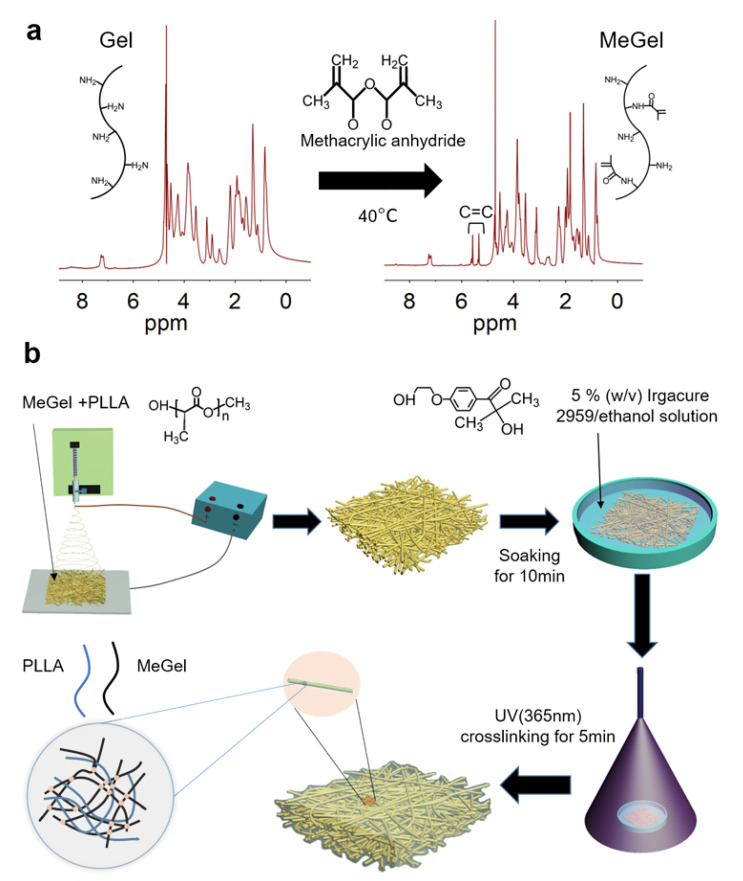
(**a**) The ^1^H-NMR of original Gel and as-prepared MeGel via the methacryloyl substitution of Gel. (**b**) The schematic of preparation process of MeGel nanofiber mats performed with a combination of electrospinning and UV crosslinking.

**Figure 2:**
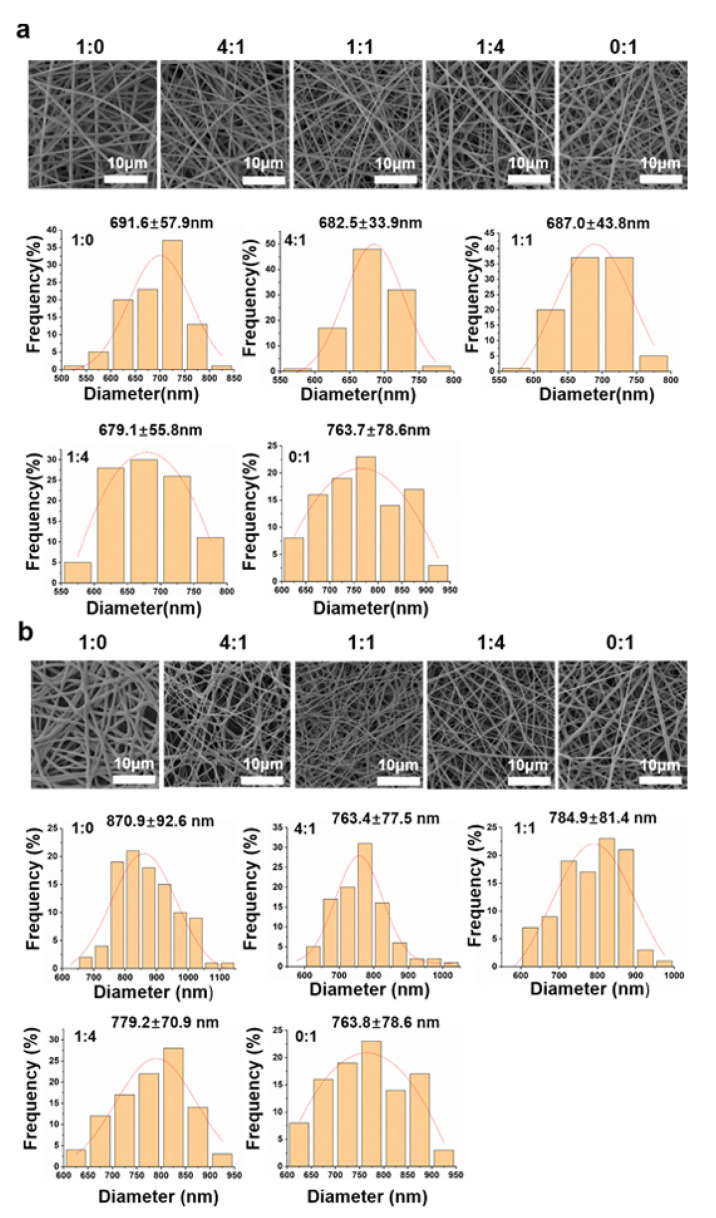
The SEM images and the frequency of the fiber diameter distribution of nanofiber mats with different MeGel/PLLA weight ratios, i.e., MeGel/PLLA = 1:0, 4:1, 1:1, 1:4, and 0:1: (**a**) before UV crosslinking and (**b**) after UV crosslinking.

The MeGel used in this study was synthesized via the methacryloyl substitution of Gel, and the substitution degree was calculated with ^1^H NMR analysis (Figure 1a). The results showed that the degree of methacryloyl substitution was ~44%. A series of nanofiber mats were designed and manufactured by first electrospinning different mass ratios of MeGel/PLLA blends in HFIP. The electrospun mats were then immersed in a 5% (*w*/*v*) Irgacure 2959/ethanol solution for 10 min and crosslinked for 5 min through exposure to UV light. The morphology of un-crosslinked and crosslinked nanofiber mats from pure MeGel, diverse MeGel/PLLA blends, and pure PLLA nanofiber mats was visualized by SEM observation (Figure 2a,b). All these un-crosslinked and crosslinked nanofiber mats exhibited uniform and bead-free fibrous morphologies, demonstrating the good electrospinnability of all MeGel/PLLA compositions. Importantly, the MeGel nanofiber mats were demonstrated to better maintain their fibrous structure after UV crosslinking treatment. The statistical analysis showed that the PLLA nanofiber mats displayed an average fiber diameter of 763.7 ± 78.6 nm, which was significantly larger than the other four un-crosslinked nanofiber mats, i.e., un-crosslinked MeGel mats (691.6 ± 57.9 nm; *p* < 0.001), un-crosslinked 4:1 MeGel/PLLA mats (682.5 ± 33.9 nm; *p* < 0.001), un-crosslinked 1:1 MeGel/PLLA mats (687.0 ± 43.8 nm; *p* < 0.001), and un-crosslinked 1:4 MeGel/PLLA mats (679.1 ± 55.8 nm; *p* < 0.001) (Figure 2a). Moreover, the statistical analysis indicated that no significant differences in the fiber diameter were present among the un-crosslinked 4:1 MeGel/PLLA mats, un-crosslinked 1:1 MeGel/PLLA mats, un-crosslinked 1:4 MeGel/PLLA mats, and un-crosslinked MeGel mats. The obviously increased fiber diameter of PLLA mats was mainly due to the significant change of spinning solution properties. The statistical analysis also showed that the crosslinked MeGel nanofiber mats displayed an average fiber diameter of 870.9 ± 92.6 nm, which was significantly larger than the other four nanofiber mats, i.e., crosslinked 4:1 MeGel/PLLA mats (763.4 ± 77.5 nm; *p* < 0.001), crosslinked 1:1 MeGel/PLLA mats (784.9 ± 81.4 nm; *p* < 0.001), crosslinked 1:4 MeGel/PLLA mats (779.2 ± 70.9 nm; *p* < 0.001), and PLLA mats (763.8 ± 78.6 nm; *p* < 0.001) (Figure 2b). Moreover, the statistical analysis indicated that no significant differences in the fiber diameter were present among the 4:1 MeGel/PLLA mats, 1:1 MeGel/PLLA mats, 1:4 MeGel/PLLA mats, and PLLA mats. It was also found that the crosslinked MeGel/PLLA nanofiber mats exhibited obviously larger fiber diameters in comparison to the corresponding un-crosslinked controls, probably due to the swelling behavior of MeGel component during UV crosslinking and the subsequent washing process.

### 3.2. FTIR and XRD Analysis of MeGel/PLLA Nanofiber Mats

**Figure 3:**
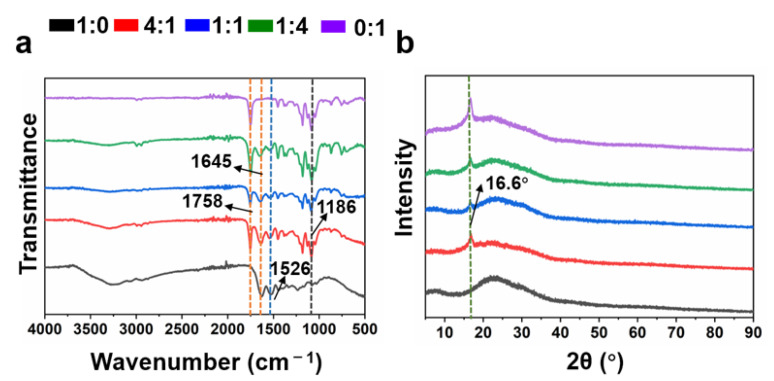
(**a**) The FTIR spectrum and (**b**) XRD spectrum of different crosslinked MeGel/PLLA nanofiber mats (MeGel/PLLA = 1:0, 4:1, 1:1, 1:4, and 0:1).

FTIR analysis was utilized to identify the functional groups and interactions of the MeGel and PLLA polymers within the fibers (Figure 3a). The PLLA nanofiber mats presented several characteristic peaks originating from the PLLA polymer, which mainly included ~1758 cm^−1^ (C=O stretching) and ~1186 cm^−1^ (–C–O stretching) [5,7]. The crosslinked MeGel nanofiber mats exhibited several characteristic peaks responsible for MeGel polymers, mainly ~1645 cm^−1^ (C=O stretching of amide I) and ~1526 cm^−1^ (N–H bending of amide II) [39]. Importantly, the characteristic peaks for the crosslinked MeGel/PLLA nanofiber mats associated with every polymer component were clearly identified. The results also showed that all the peak positions of crosslinked MeGel/PLLA nanofiber mats exhibited no obvious shifting and the generation of no new functional groups, indicating that only simple blend processes occurred in the fibers with different MeGel/PLLA weight ratios.

The XRD patterns of crosslinked MeGel, 4:1 MeGel/PLLA, 1:1 MeGel/PLLA, 1:4 MeGel/PLLA, and PLLA nanofiber mats are shown in Figure 3b. The PLLA nanofiber mats exhibited a wide peak at roughly 16.6° corresponding to (200) crystal plain originating from the PLLA. The MeGel nanofiber mats displayed a taro peak around 20°. When the different mass ratios of MeGel and PLLA were combined into nanofibers, the MeGel/PLLA nanofibers also exhibited a crystallization peak at roughly 16.6° [40].

### 3.3. Hydrophilicity, Swellability, Swelling Kinetics and Air Permeability of MeGel/PLLA Nanofiber Mats

**Figure 4:**
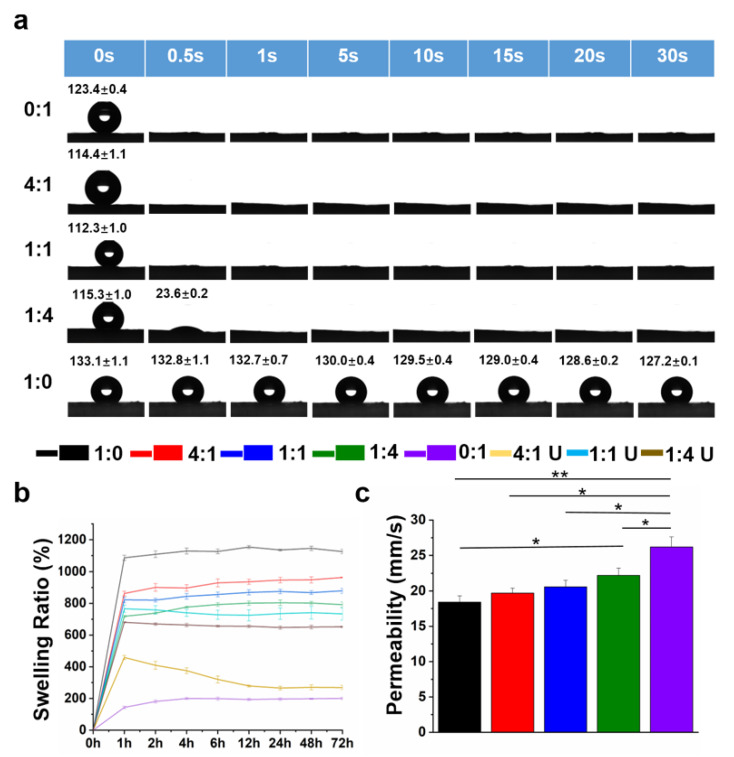
(**a**) Images of the water contact angle, (**b**) the swelling ratio, and (**c**) the air permeability of different MeGel/PLLA nanofiber mats after UV crosslinking (MeGel/PLLA = 1:0, 4:1, 1:1, 1:4, and 0:1) (n = 5; * *p* < 0.05, ** *p* < 0. 01). The 4:1 U, 1:1 U, and 1:4 U in (**b**) stand for the swelling ratio of un-crosslinked nanofiber mats with the MeGel/PLLA = 4:1, 1:1, and 1:4, respectively.

The water contact angle was assessed to determine the surface hydrophilicity and wettability of the crosslinked nanofiber mats (Figure 4a). The results showed that the PLLA nanofiber mats presented obvious hydrophobicity, with a water contact angle of ~127°. In comparison, the introduction of the MeGel component into nanofiber mats significantly improved the surface hydrophilicity. For instance, the 1:4 MeGel/PLLA nanofiber mats with the lowest MeGel content exhibited a water contact angle of 0°, and the water droplet was found to be rapidly spread and totally absorbed on the 1:4 MeGel/PLLA nanofiber mats in 1 s. Basar et al. found that the hydrophilicity of electrospun Gel/PCL nanofiber mats was significantly higher than that of PCL nanofiber mats (<0.1° vs. ~116.7°, respectively) [41]. Chen et al. demonstrated that the hydrophilicity of the electrospun nanofiber mats was significantly improved by the addition of Gel (~32°) in comparison to PLLA alone (~134°) [40] Therefore, the introduction of Gel or MeGel into a hydrophobic scaffold or matrix is an effective way to improve hydrophilicity.

An ideal wound dressing material should not only offer a moist environment to avoid wound dehydration but also have the capability to absorb excessive wound exudates [42]. The water uptake capacity of different MeGel/PLLA nanofiber mats before and after UV crosslinking was also evaluated. As shown in Supplemental Video S1, some parts of the un-crosslinked MeGel mats rapidly dissolved and the structure of mats was totally broken once deionized water was added. In comparison, the crosslinked electrospun MeGel mats could better remain the mat-like structure in the deionized water, as shown in Supplemental Video S2. The water uptake capacity of the un-crosslinked MeGel mats could be not detected. In comparison, the crosslinked MeGel nanofiber mats presented a significantly high swelling ratio of 1135.2 ± 16.0% (Figure 4b). It was found that decreasing the MeGel/PLLA weight ratio could improve the swelling ratio of un-crosslinked MeGel/PLLA nanofibrous mats to some extent. This phenomenon could be explained that the un-crosslinked MeGel component was water-dissolvable, and increasing the PLLA component could hinder the immediate dissolution of the MeGel component. Interestingly, a totally opposite phenomenon was observed for the crosslinked MeGel/PLLA nanofibrous mats. The results showed that the crosslinked nanofiber mats with higher amounts of MeGel presented significantly higher swelling ratios in comparison to the crosslinked nanofiber mats with lower amounts MeGel. For example, the swelling ratio of crosslinked nanofiber mats following 1 h of incubation increased from 143.6 ± 7.4% to 803.8 ± 5.2%, 875.0 ± 17.1%, 946.9 ± 14.4%, and 1135.2 ± 16.0% when the MeGel/PLLA mass ratio increased from 0:1 to 1:4, 1:1, 4:1, and 1:0, respectively. This phenomenon could be explained by the presence of hydrophilic groups including hydroxyl and amine in the MeGel macromolecules, which notably enhanced the swelling ratio of crosslinked nanofiber mats.

**Figure 5:**
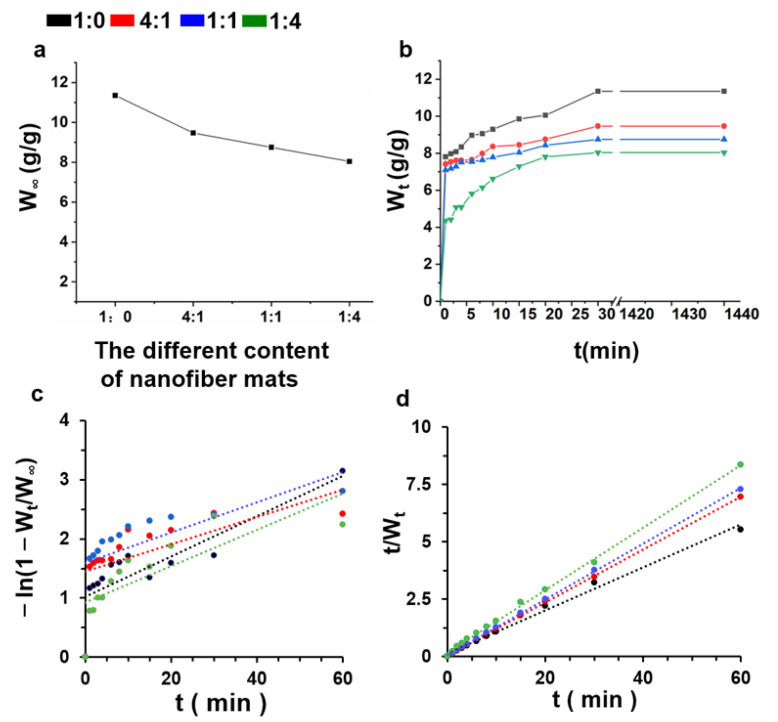
(**a**) The equilibrium swelling ratio, (**b**) the swelling ratio at different times, (**c**) first-order fitting lines, and (**d**) second-order fitting lines of different crosslinked MeGel/PLLA nanofiber mats (MeGel/PLLA = 1:0, 4:1, 1:1, and 1:4).

Figure 5a,b shows the equilibrium swelling ratio and swelling kinetics of the MeGel-containing nanofibrous mats after UV crosslinking, respectively. The swelling ratio of all MeGel-containing samples rapidly increased at the initial time of soaking, and then a slower swelling process occurred until a swelling equilibrium was reached. The osmotic pressure difference between the inside and outside of MeGel-containing mats resulted in the rapid entry of water molecules into the mats after the initial 2 min. It was also found that the nanofiber mats with higher MeGel contents presented higher swelling ratios and shortened swelling equilibrium times due to the fact that the increasing of the MeGel component made it easier for the water molecules to enter the nanofiber mats. Two classical models, i.e., first-order kinetics and second-order kinetics, were employed to verify the swelling kinetics of the MeGel-containing samples. As shown in Figure 5c, the swelling kinetics of all samples did not fit the first-order kinetics. However, the swelling kinetics of all samples could fit well with Schott second-order kinetics, as shown in Figure 5d. The second-order fitting lines of 1:0, 4:1, 1:1, and 1:4 MeGel/PLLA nanofiber mats were y = 0.0938x + 0.1408, y = 0.1153x + 0.0512, y = 0.1217x + 0.0528, and y = 0.1363x + 0.1708, respectively.

An appropriate air permeability is of significant importance in wound dressing materials to maintain the comfort of the wound site and accelerate the healing process [43]. As shown in Figure 4c, the air permeability of crosslinked MeGel, 4:1 MeGel/PLLA, 1:1 MeGel/PLLA, 1:4 MeGel/PLLA, and PLLA nanofiber mats was 18.7 ± 0.5%, 19.1 ± 0.3%, 20.6 ± 0.4%, 18.9 ± 1.9%, and 26.2 ± 1.4%, respectively. This phenomenon could be explained by the increase of fiber diameter and the decrease of the fiber gap after crosslinking with the increase of MeGel component in the nanofiber mats.

### 3.4. Mechanical Properties of MeGel/PLLA Nanofiber Mats

**Figure 6:**
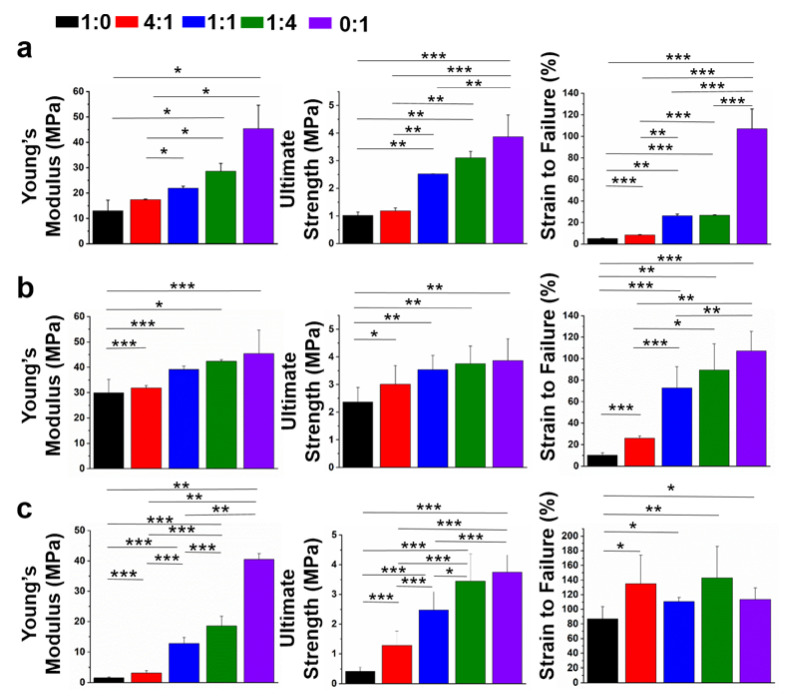
(**a**) The mechanical properties of un-crosslinked nanofiber mats with different MeGel/PLLA mass ratios (n = 5; * *p* < 0.05, ** *p* < 0.01, *** *p* < 0.001). The mechanical properties of crosslinked nanofiber mats with different MeGel/PLLA mass ratios in (**b**) the dry condition dry and (**c**) the wet condition (n = 5; * *p* < 0.05, ** *p* < 0.01, *** *p* < 0.001).

Appropriate mechanical properties are also key factors for the construction of wound dressing materials, which should enable the maintenance of their structural stability to protect wound sites [44]. The mechanical properties of MeGel-containing nanofibrous scaffolds with and without crosslinking, as well as PLLA nanofiber mats, were determined in a dry condition (Figure 6a,b). The Young’s modulus for un-crosslinked MeGel, 4:1 MeGel/PLLA, 1:1 MeGel/PLLA, 1:4 MeGel/PLLA, and PLLA nanofiber mats in the dry condition was 13.0 ± 4.2 MPa, 17.4 ± 0.2 MPa, 21.9 ± 0.8 MPa, 28.6 ± 3.1 MPa, and 45.4 ± 9.3 MPa, respectively. As comparison, the Young’s modulus for the crosslinked MeGel, 4:1 MeGel/PLLA, 1:1 MeGel/PLLA, 1:4 MeGel/PLLA nanofiber mats in the dry condition was 30.0 ± 5.3 MPa, 31.9 ± 0.9 MPa, 39.1 ± 1.1 MPa, 42.3 ± 0.7 MPa, respectively. The ultimate strength was 1.0 ± 0.1 MPa for un-crosslinked MeGel, 1.2 ± 0.1 MPa for un-crosslinked MeGel/PLLA (4:1), 2.5 ± 0.1 MPa for un-crosslinked MeGel/PLLA (1:1), 3.1 ± 0.2 MPa for un-crosslinked MeGel/PLLA (1:4), and 3.9 ± 0.8 MPa for PLLA all in the dry situation. In contrast, the ultimate strength was 2.4 ± 0.5 MPa for crosslinked MeGel, 3.0 ± 0.7 MPa for crosslinked MeGel/PLLA (4:1), 3.5 ± 0.5 MPa for crosslinked MeGel/PLLA (1:1), 3.8 ± 0.6 MPa for crosslinked MeGel/PLLA (1:4) all in the dry situation. The results showed that all the un-crosslinked MeGel-containing nanofiber mats presented obviously decreased mechanical properties compared to the corresponding crosslinked mats all in the dry condition. Importantly, the Young’s modulus of our crosslinked MeGel/PLLA nanofiber mats was all more than 30 MPa, which were significantly higher compared to the Gel/PLA un-crosslinked nanofiber mats [45]. For instance, the Young’s modulus of Gel/PLA nanofiber mats fabricated by Chen et al. was only in the range of 0.2–1.37 MPa [40]. Taken together, the UV crosslinking process was demonstrated to significantly improve the structural stability and mechanical properties of MeGel/PLLA nanofiber mats.

The mechanical properties of crosslinked MeGel-containing nanofiber mats and PLLA nanofiber mats were also determined in a wet condition (Figure 6c). The Young’s modulus of crosslinked MeGel, 4:1 MeGel/PLLA, 1:1 MeGel/PLLA, 1:4 MeGel/PLLA, and PLLA nanofiber mats in the wet condition was 1.5 ± 0.3, 3.2 ± 0.7, 12.8 ± 2.0, 18.5 ± 3.3, and 40.5 ± 1.9 MPa, respectively. The ultimate strength was 2.4 ± 0.5 MPa for crosslinked MeGel, 3.0 ± 0.7 MPa for crosslinked MeGel/PLLA (4:1), 3.5 ± 0.5 MPa for crosslinked MeGel/PLLA (1:1), 3.8 ± 0.6 MPa for crosslinked MeGel/PLLA (1:4), and 3.9 ± 0.8 MPa for PLLA in the wet situation. The results showed that all the nanofiber mats presented obviously decreased mechanical properties in the wet condition in comparison to those the dry condition. Moreover, the mechanical properties of nanofiber mats significantly decreased with the increase of the MeGel/PLLA weight ratio, especially in the wet condition. 

### 3.5. Biocompatibility of MeGel/PLLA Nanofiber Mats

**Figure 7:**
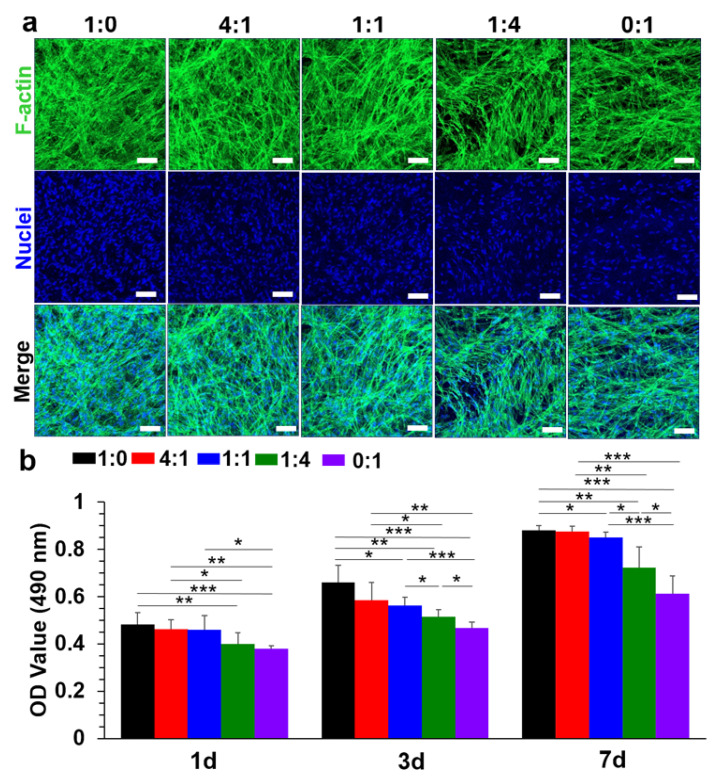
(**a**) The F-actin (green) and nuclei (blue) immunofluorescent staining of human dermal fibroblasts cultured on different crosslinked MeGel/PLLA nanofiber mats at day 7. Scale bars = 100 μm. (**b**) The MTT assay results of human dermal fibroblasts cultured on different crosslinked MeGel/PLLA nanofiber mats over 7 days (n = 5; * *p* < 0.05, ** *p* < 0.01, *** *p* < 0.001).

Dermal fibroblasts are sone of the most important cell phenotypes in human skin, and they play a key role during the wound healing process. One of the necessary requirements for wound dressing materials is the ability to provide an appropriate structure-supportive microenvironment to support the cell behaviors of dermal fibroblasts [46]. Therefore, human dermal fibroblasts were selected to preliminarily investigate cell viability, growth, and proliferation behaviors on the different crosslinked MeGel/PLLA nanofiber mats. An F-actin and DAPI double-staining method was utilized to visualize the cytoskeleton (green) and nuclei (blue) of human dermal fibroblasts seeded on the five different nanofiber mats after 7 days of culture (Figure 7a). The results showed that the human dermal fibroblasts presented spindle-like morphology on all five different nanofiber mats, and the cells adhered and grew well on the five different nanofiber mats.

The proliferation behavior of human dermal fibroblasts seeded on the five different crosslinked nanofiber mats was further determined using an MTT assay (Figure 7b). The results showed that the OD values of the five different nanofiber mats were obviously different. The MeGel nanofiber mats exhibited the highest OD value and the PLLA nanofiber mats presented lowest OD value at days 1, 3, and 7, and the OD value improved with the increase of the MeGel/PLLA weight ratio. These results indicated that the introduction of a MeGel bioactive component was assuredly beneficial for cell adhesion, growth, and proliferation, and the cell proliferation capacity notably enhanced with the increase of the MeGel/PLLA mass ratio.

Lobo et al. reported that the introduction of a MeGel bioactive component into PCL nanofiber mats significantly promoted the attachment and proliferation of human osteoblasts [39]. Niu et al. found that the Gel/PLLA nanofiber mats notably promoted the elongation and proliferation of Schwann cells [47]. The significantly enhanced cell attachment and proliferation on the Gel or MeGel-containing scaffolds found here probably originated from the great hydrophilicity and ECM-like hydrogel characteristics. Previous studies have shown that the surface hydrophilicity of one scaffold presents a positive effect on cell attachment and proliferation [48]. Moreover, some previous studies demonstrated that the Gel component, originating from collagen (a major component of most soft tissues), could better mimic the biological properties of native ECM and thus significantly promote cell adhesion and growth [28,29,41]. Overall, our crosslinked MeGel/PLLA nanofiber mats offered a favorable microenvironment to promote the attachment, growth, and proliferation of human dermal fibroblasts, so they have potential as candidates for wound dressing material applications. It should be noted that the animal model validation of these MeGel/PLLA nanofiber mats is required in future work.

## 4. Conclusions

In this study, a series of different nanofiber mats were manufactured by blending MeGel and PLLA polymers with different weight ratios and the UV crosslinking process, and the nanofiber mats presented uniform and bead-free fibrous morphology. The results demonstrated the feasibility for adjusting the surface hydrophilicity, wettability, swellability, swelling kinetics, air permeability, mechanical, and biological properties of as-obtained nanofibrous mats by blending MeGel and PLLA components. This study provides meaningful guidance for the design and construction of MeGel/PLLA blend nanofiber mats for potential wound dressing material research and application.

## Data Availability

The data presented in this study are available in the article or Supplementary material.

## Supplementary Materials

The following are available online at https://www.mdpi.com/article/10.3390/nano12010006/s1, Video S1: Un-crosslinked MeGel mats; Video S2: The crosslinked electrospun MeGel mats.
